# Treatment Strategies and Outcomes for Single-Site Recurrent Pancreatic Ductal Adenocarcinoma After Curative-Intent Surgery: Local Therapies are Associated with Longer Survival in Selected Cases: A Systematic Review and Meta-analysis

**DOI:** 10.1245/s10434-026-19939-w

**Published:** 2026-06-16

**Authors:** Aya Maekawa, Giampaolo Perri, Danhui Heo, Giulia Cemin, Anouk Janine van Bodegraven, Clarissa De Nardi, Agata Grochowska, Umberto Cillo, Giovanni Marchegiani

**Affiliations:** 1 Department of Surgical, Oncological and Gastroenterological Sciences, DiSCOG, University of Padova, Padova, Italy; 2 Department of Hepatobiliary and Pancreatic Surgery, Institute of Science Tokyo, Tokyo, Japan; 3 Doctoral School of Experimental and Preventive Medicine, Albert Szent-Györgyi Medical School, University of Szeged, Szeged, Hungary; 4 Department of Surgery, Amsterdam UMC location VUmc, University of Amsterdam, Amsterdam, The Netherlands; 5 Department of General and Transplant Surgery, Medical University of Lodz, Łódź, Poland

## Abstract

**Background:**

After curative-intent pancreatectomy for pancreatic ductal adenocarcinoma (PDAC), disease recurrence occurs almost invariably. Evidence guiding treatment of recurrence remains limited and may differ across single-site recurrence patterns.

**Methods:**

Scopus, MEDLINE, and the Cochrane Library were systematically searched for observational studies of adults with recurrent PDAC reporting liver-only, lung-only, or locoregional recurrence and post-recurrence treatments. Hazard ratio (HR) meta-analyses were performed to compare survival outcomes between treatment strategies for each recurrence location with additional descriptive survival summaries.

**Results:**

The inclusion criteria were met by 22 studies, with 17 included in the quantitative synthesis. Pattern-specific analyses favored selected local therapies. For liver-only recurrence, local therapy was associated with longer overall survival than chemotherapy alone (HR 0.26, 95% confidence interval [CI] 0.14–0.49), mainly due to surgical resection (HR 0.18; 95% CI 0.11–0.31). Ablation did not reach statistical significance. In lung-only recurrence, surgical resection was associated with longer survival than conventional management (HR 0.35; 95% CI 0.26–0.48). For locoregional recurrence, resection was associated with longer survival (HR 0.52; 95% CI 0.38–0.72). Stereotactic body radiotherapy outcomes from three small series were summarized descriptively.

**Conclusions:**

Pancreatic ductal adenocarcinoma recurrence outcomes differ significantly by recurrence pattern. Across liver-only, lung-only, and locoregional recurrence, selected patients undergoing local therapy had longer survival than those managed conventionally. Although current observational data do not confirm broad efficacy for specific methods, these results support recurrence pattern-specific prospective evaluation to define optimal site-tailored strategies.

**Supplementary Information:**

The online version contains supplementary material available at 10.1245/s10434-026-19939-w.

Pancreatic ductal adenocarcinoma (PDAC) remains one of the most aggressive malignancies, with a 5-year survival rate of approximately 10% despite advances in surgical techniques and systemic treatments.^1,2^ Even after curative-intent resection, 70–80% of patients experience recurrence already within 2 years.^2,3^ Recurrence may occur locally in the remnant pancreas or surrounding tissues, or as distant metastases, most commonly in the liver, followed by the lungs and peritoneum.^4–7^ These recurrence patterns differ not only in prognosis but also in potential treatment strategies.

Systemic chemotherapy remains the standard treatment for recurrent PDAC.^8–10^ Modified fluorouracil, leucovorin, irinotecan, and oxaliplatin (FOLFIRINOX) or gemcitabine combined with nab-paclitaxel is commonly used, either as reintroduction or after modification of prior neoadjuvant or adjuvant treatments.^11^ Current guidelines provide limited recurrence-specific, evidence-based algorithms.^12–15^ Systemic therapy remains the default approach, whereas local therapies such as surgical resection, radiotherapy, ablation, and multimodal approaches are considered for highly selected patients with isolated local recurrence or oligometastatic disease.^4,16–18^

Recurrent PDAC also differs from “de novo” metastatic disease.^19^ Recurrence develops after a disease-free interval, often after prior systemic therapy, and often is detected under surveillance at an earlier or low-burden stage. Despite these differences, many studies and clinical practices continue to classify both entities as “metastatic PDAC,” obscuring potential biologic and therapeutic distinctions.

Although several studies and reviews have described recurrence patterns and prognostic factors,^20–23^ an integrated synthesis across treatment methods for major single-site recurrence patterns remains limited. To address this gap, we conducted a systematic review of treatment strategies for recurrent PDAC after curative-intent resection, focusing on single-site recurrence.

## Methods

### Protocol and Reporting

This systematic review and meta-analysis followed a predefined protocol registered in PROSPERO (CRD420251082788) and was conducted in accordance with the Preferred Reporting Items for Systematic Reviews and Meta-Analyses (PRISMA)^24^ and the Meta-analysis Of Observational Studies in Epidemiology (MOOSE) guidelines.^25^

### Eligibility Criteria

Observational studies reporting outcomes after recurrence of PDAC in adults after curative-intent pancreatectomy were included. Eligible studies evaluated at least one predefined recurrence pattern of liver-only, lung-only, or locoregional recurrence, and reported outcomes of surgical resection, ablative procedures, chemotherapy, radiotherapy, and other local therapies administered after recurrence. Peritoneal recurrence was not included because it is frequently multifocal and inconsistently reported as a distinct recurrence pattern, limiting meaningful pattern-specific comparisons of local therapies.

Comparative studies were required to include a concurrent systemic or non-surgical therapy cohort. Single-arm studies were included solely for descriptive synthesis and excluded from comparative analyses. Case reports, reviews, conference abstracts, non-human studies, and cohorts with mixed pancreatic malignancies or metachronous new primary tumors were excluded unless PDAC-specific data were extractable or non-PDAC histology represented less than 10%.

### Search Strategy and Study Selection

A systematic search of MEDLINE, Scopus, and the Cochrane Library from January 2010 to July 2025 was performed using predefined terms covering PDAC, recurrence, treatment, and survival (Supplementary Material). The reference lists of included articles and relevant reviews were manually screened to identify additional eligible studies.

**Fig. 1:**
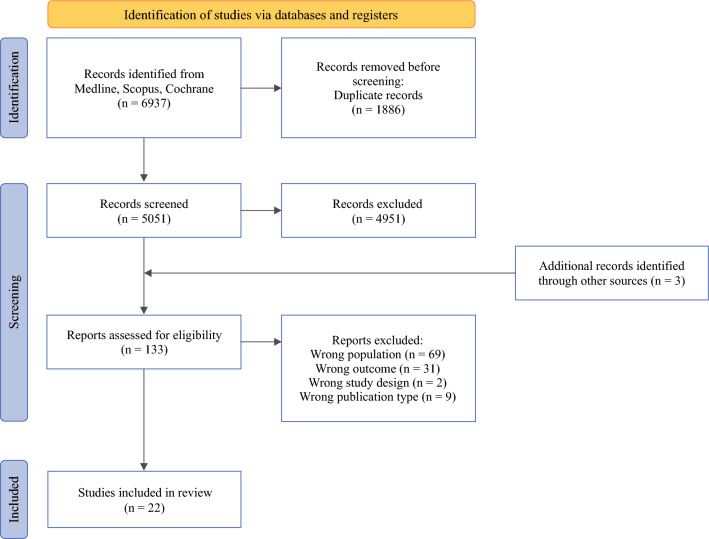
PRISMA diagram of the systematic review

Two reviewers (A.M. and D.H.) independently screened titles and abstracts. Full-text eligibility was assessed by A.M., G.C., A.vB., and C.DN. Discrepancies were resolved through discussion. The study selection process is summarized in the PRISMA flow diagram depicted in Fig. 1.

### Definitions

Curative-intent surgery was defined as resection of pathologically confirmed localized PDAC with R0 or R1 margins. Recurrence was defined as radiologic, histologic, or unequivocal biochemical evidence of PDAC after a disease-free interval. Recurrence patterns were categorized as liver-only, lung-only, or locoregional, with locoregional including lesions in the remnant pancreas, pancreatic bed, or regional lymph nodes.

Systemic therapy included cytotoxic chemotherapy, with or without radiotherapy, administered with disease-controlling intent. Local therapy included surgical resection, radiofrequency ablation (RFA), or radiotherapy delivered to the recurrence site. Overall survival (OS) was defined per study and, when available, was preferentially reported as time from initial pancreatectomy to death. When studies reported survival from recurrence, it was analyzed as post-recurrence survival (PRS), defined as time from recurrence to death from any cause. For stereotactic body radiotherapy (SBRT) cohorts, progression-free survival (PFS) was extracted as reported.

### Data Extraction

A standardized data extraction form was used to collect study characteristics, recurrence patterns, treatment details, and survival outcomes. Due to variability in OS definitions and time origins across studies, survival after recurrence was extracted only when it could be reliably obtained or harmonized.

Hazard ratios (HRs) were extracted when directly reported, estimated from summary statistics, or reconstructed from Kaplan–Meier curves. When survival data were not explicitly reported, time-specific survival proportions were extracted from published tables or digitized from Kaplan–Meier curves using WebPlotDigitizer, version 5.2: Ankit Rohatgi, Pacifica, CA, USA.^26^ Information on local therapy selection criteria was extracted when reported and is summarized descriptively in Tables 1, 2, 3.

**Table 1:** Reported selection criteria for local therapy in liver-only recurrence

Author, Year	Local therapy	Maximum no. of lesions	Size criteria (cm)	Chemotherapy duration (months)	Chemotherapy response required
Schwarz 2020	Surgical resection	NR	NR	NR	No
Mitsuka 2020	Surgical resection	≤ 3	NR	NR	No
Shibata 2023	Surgical resection	1	NR	≥ 6	Yes
Lee 2020	RFA	≤ 5	≤ 5	NR	Yes
Wang 2023	RFA	NR	NR	NR	No
Kim 2025	RFA or surgical resection	≤ 2	NR	NR	No

*NR* not reported, *RFA* radiofrequency ablation

### Outcomes

The primary outcomes were OS and PRS, stratified by recurrence pattern. Secondary outcomes included treatment patterns across recurrence types. For non-comparative SBRT cohorts, OS and PFS were summarized descriptively using time-specific survival proportions when available.

### Risk of Bias Assessment

The methodologic quality of the included studies was evaluated using the Risk of Bias in Non-randomized Studies of Interventions (ROBINS-I) tool.^27^ This tool evaluates the risk of bias across seven domains: confounding, participant selection, intervention classification, deviations from intended interventions, missing data, outcome measurement, and selection of the reported result. A “traffic light” plot was generated to visualize the risk-of-bias assessment results using the robvis (Luke A. McGuinness, University of Bristol, Bristol, UK).^28^

### Statistical Analysis

Hazard ratios were pooled using inverse variance-weighting within a random-effects model on the log-transformed scale, with between-study variance estimated by restricted maximum likelihood. Pooled estimates and 95% confidence intervals (CIs) were calculated using the Wald method, and heterogeneity was assessed using the *I*^2^ statistic.

Survival outcomes were summarized descriptively, without meta-analytic pooling, to characterize treatment-specific survival patterns. Time-specific survival proportions were calculated at prespecified intervals using cohort size-weighted estimates for visual comparison. Only studies reporting the relevant time point contributed to each estimate, and missing values were not imputed.

**Table 2:** Reported selection criteria for local therapy in lung-only recurrence

Author, Year	Local therapy	Maximum number of lesions	Laterality	Chemotherapy duration	Chemotherapy response required
Groot 2019	Surgical resection	NR	NR	NR	NR
Kurahara 2020	Surgical resection	NR	NR	NR	NR
Safi 2021	Surgical resection	NR	NR	NR	NR
Mashiko 2021	Surgical resection	NR	NR	NR	Yes (when performed)
Homma 2022	Surgical resection	NR	NR	NR	NR
Stuart 2023	Surgical resection	NR	NR	NR	NR
Arnaoutakis 2011	Surgical resection	NR	NR	NR	NR
Otsuka 2020	Surgical resection	≤ 3	NR	NR	NR
Shimizu 2020	Surgical resection	NR	NR	NR	NR
Ilmer 2019	Surgical resection	NR	NR	NR	NR

*NR* not reported

**Table 3:** Reported selection criteria for local therapy in locoregional recurrence

Author, year	Local therapy	Anatomic resectability stated	Chemo response required
Strobel 2013	Surgical resection	CT-based; SMA or celiac trunk encasement defined as unresectable	NR
Yamada 2018	Surgical resection	No major vascular invasion	NR
Kim 2019	Surgical resection	R0 resection possibility	NR
Safi 2021	Surgical resection	NR	NR
Comito 2017	SBRT	Isolated local recurrence, < 5 cm, no distant metastases	NR
Li 2020	SBRT	NR	NR
Reddy 2022	SBRT	NR	NR

*CT* computed tomography, *SMA* superior mesenteric artery, *NR* not reported, *SBRT* stereotactic body radiation therapy

Subgroup analyses were performed to examine differences in survival patterns by treatment method within a consistent analytical framework. Local therapy methods were analyzed separately when comparative data were available. Non-comparative SBRT cohorts were summarized descriptively and not pooled with surgical resection studies because of differences in study design, treatment intent, and endpoint reporting. When treatment definitions varied across studies, subgroup classifications were adapted to permit further stratification within broader local or conventional treatment categories.

Publication bias and small-study effects were assessed visually using funnel plots for the main comparative analyses. Because each comparison included fewer than 10 studies, funnel plot interpretation was considered exploratory, and formal tests for asymmetry were not performed.

All analyses were conducted using R (version 4.5.1; R Foundation for Statistical Computing, Vienna, Austria) and Review Manager 5.4 (The Cochrane Collaboration, Copenhagen, Denmark).

## Results

### Study Selection

A total of 6937 records were identified through database searching. After removal of duplicates, 5051 records remained. After title and abstract screening, 133 articles underwent full-text assessment, and 22 studies met the inclusion criteria. Of these studies, 17 provided sufficient data for quantitative synthesis (Fig. 1).

### Study Characteristics

The 22 included studies, published between 2011 and 2025, originated from Asia, Europe, and North America. All were observational cohort studies. Comparators were typically systemic chemo(radio)therapy or best supportive care (BSC), and a subset of studies reported adjusted analyses using multivariable models. In particular, the SBRT series frequently lacked formal control groups. The main characteristics of the included studies are summarized in Tables S1–3.

### Primary Comparative Analyses

#### Liver-Only Recurrence

**Fig. 2:**
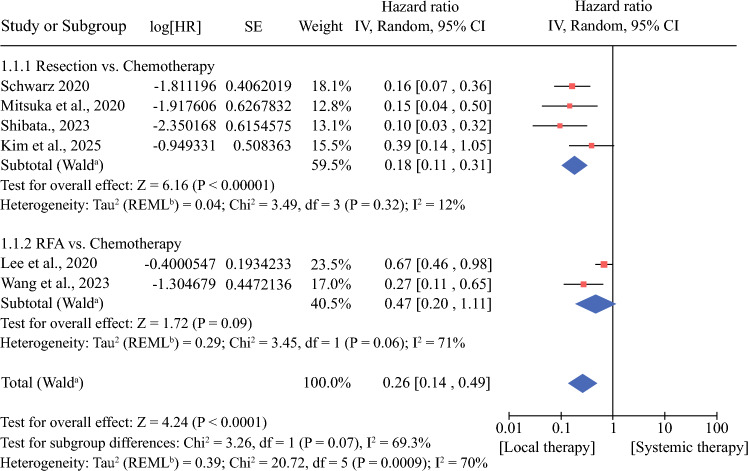
Liver-only recurrence: local therapy versus chemotherapy. Forest plot of hazard ratios for overall survival comparing liver-directed local therapy (resection or ablation) with chemotherapy

In liver-only recurrence, six comparative studies including 350 patients reported OS for local therapy (liver resection or RFA) versus systemic chemotherapy (Fig. 2).^29–34^ Of these 350 patients, 133 received local therapy, and 217 received chemotherapy or conventional management. Local therapy was associated with longer OS than systemic therapy (HR 0.26; 95% CI 0.14–0.49), although substantial heterogeneity was observed across studies (*I*^2^ = 69.3%). In subgroup analyses, surgical resection showed a consistent association with longer survival and low heterogeneity (HR 0.18; 95% CI 0.11–0.31; *I*^2^ = 12%), whereas RFA did not reach statistical significance and showed substantial heterogeneity (HR 0.47; 95% CI 0.20–1.11; *I*^2^ = 71%). The difference between subgroups did not reach statistical significance (*p* = 0.07).

In descriptive synthesis, cohort size-weighted time-specific OS estimates were directionally consistent with the pooled HRs, with a higher 3-year OS after liver resection than after chemotherapy (Fig. S1).

#### Lung-Only Recurrence

**Fig. 3:**
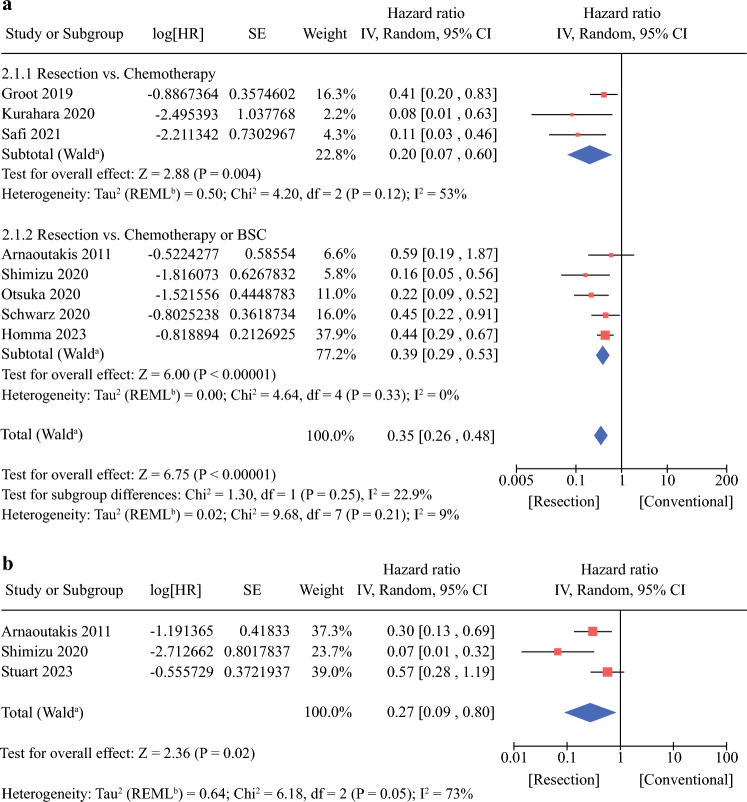
Lung recurrence: resection versus conventional management. **a** Post-recurrence survival. **b** Overall survival

In lung-only recurrence, 10 studies included 429 patients, 125 of whom underwent surgical resection (Table S1).^4,35–43^ Among 418 patients in nine comparative studies, 114 received surgical resection, and 304 received conventional management, mainly chemo(radio)therapy, BSC, or non-resection approaches. Eight comparative studies provided extractable effect estimates for PRS and were pooled in the meta-analysis (Fig. 3).

Surgical resection was associated with longer PRS than conventional management (HR 0.35; 95% CI 0.26–0.48; *I*^2^ = 9%). The association remained consistent across studies comparing surgical resection with chemotherapy (HR 0.20; 95% CI 0.07–0.60; 3 studies) and surgical resection with chemotherapy or BSC (HR 0.39; 95% CI 0.29–0.53; 5 studies). Three studies reported OS, and pooled analysis also favored surgical resection (HR 0.27; 95% CI 0.09–0.80), although heterogeneity was substantial (*I*^2^ = 73%; Fig. 3).

Descriptive synthesis indicated that cohort size-weighted 2-year PRS was higher after surgical resection than after non-surgical management (73.1% vs 36.1%) (Fig. S2).

#### Locoregional Recurrence

**Fig. 4:**
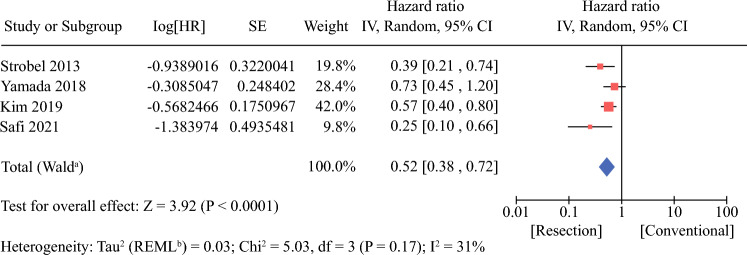
Locoregional recurrence: resection versus conventional management. Forest plot of hazard ratios for post-recurrence survival

Four comparative studies, including 233 patients, evaluated surgical resection for isolated locoregional recurrence versus conventional management.^7,36,44,45^ Of these 233 patients, 167 underwent resection, and 66 received conventional non-surgical management. Pooled analysis showed an association between resection and longer survival (HR 0.52; 95% CI 0.38–0.72; *I*^2^ = 31%) (Fig. 4). The surgical procedures were highly heterogeneous, ranging from remnant pancreatectomy to excision of paraaortic/paracaval and mesenteric-axis area recurrences, and occasionally multivisceral resections (gastrectomy, colectomy, diaphragmatic resection, or psoas resection), reflecting substantial variability across studies. Three non-comparative SBRT cohorts, including 77 patients, were identified and synthesized descriptively.^46–48^

In descriptive synthesis, cohort size-weighted time-specific survival estimates after SBRT are summarized in Fig. S3, with 1-year OS and PFS of 58.4% and 43.4%, respectively.

### Risk of Bias

The results of the risk-of-bias assessment are shown in Fig. S4. Overall, the included studies were judged to have low to moderate risk of bias across most ROBINS-I domains, including participant selection, intervention classification, missing data, outcome measurement, and reporting. Across studies, concerns were mainly related to confounding inherent to non-randomized study designs. Among the 22 included studies, 19 were assessed as having a moderate overall risk of bias, whereas 3 were assessed as having a serious risk of bias.

Funnel plots for the main comparative analyses are shown in Fig. S5. Given the small number of studies within each recurrence pattern-specific comparison, assessment of asymmetry was exploratory and could not exclude publication bias or small-study effects.

### Reported Selection Criteria for Local Therapy

Selection criteria for local therapy were heterogeneous and often incompletely described across recurrence patterns (Tables 1–3). For liver-only recurrence, studies more frequently reported lesion number or size, whereas chemotherapy duration and response were less commonly specified. In lung-only recurrence, most studies reported surgical resection in selected patients, but rarely detailed reproducible criteria such as the maximum number of lesions, laterality, chemotherapy duration, or response. For locoregional recurrence, surgical series more often addressed anatomic resectability or technical feasibility, but rarely included biologic criteria such as chemotherapy response or disease stability. The frequent absence of reported criteria in Tables 1–3 underscores the lack of standardized reporting for local therapy eligibility in recurrent PDAC.

## Discussion

This systematic review and meta-analysis evaluated treatment strategies for single-site recurrent PDAC after curative-intent surgery, with a focus on liver-only, lung-only, and locoregional recurrence. By analyzing these recurrence patterns within a unified framework that integrates comparative HRs and time-specific survival estimates, clinically meaningful heterogeneity in survival outcomes was identified.

The association between local therapy and survival differed by recurrence site. In liver-only recurrence, the comparative evidence was driven mainly by surgical resection, and local therapy was associated with longer OS compared with systemic therapy.^29–32^ The estimate for RFA was less precise and did not reach statistical significance, consistent with limited power and heterogeneity.^33,34^ In lung-only recurrence, surgical resection was associated with longer PRS with low heterogeneity, whereas OS results were directionally consistent but more variable across studies.^4,35–41^ In locoregional recurrence, surgical resection was associated with longer survival than conventional management,^7,36,44,45^ whereas SBRT cohorts were smaller and reported descriptively.^46–48^ Across sites, these findings should be interpreted cautiously given the observational nature of the evidence and the potential for selection and residual confounding.

These site-specific findings should be interpreted in the context of recurrence after pancreatectomy, which is typically detected during surveillance and occurs after prior systemic therapy. This differs from de novo metastatic disease and may create a subset with isolated or low-burden recurrence for which local approaches could be considered. Moreover, by identifying observed associations between treatment strategy and survival within specific recurrence patterns, our synthesis helps to define where future comparative studies, ideally randomized trials where feasible, should be prioritized. Cohort size-weighted survival estimates provided a supportive context and were directionally consistent with the comparative analyses, but cross-site comparisons are indirect and should be viewed as hypothesis-generating.

The clinical meaning of these results centers on patient selection, not method-specific efficacy. Favorable hazard ratios do not imply broad applicability; they likely reflect outcomes among highly selected patients who are unlikely to be comparable with those receiving conventional treatment. In this review, biologically limited recurrence refers to disease with a limited burden and an indolent clinical course, such as isolated recurrence, a longer disease-free interval, absence of additional metastatic spread, or stability during systemic therapy. Technically manageable recurrence refers to disease that is anatomically suitable for complete resection, ablation, or focused radiotherapy with acceptable morbidity.

Therefore, pooled hazard ratios should be interpreted as estimates of association for selected patients, reflecting both patient selection and potential treatment effect rather than isolated causal effects of local therapy. Selection can inform precision oncology only if eligibility criteria are explicit, reproducible, and prospectively evaluated. Immortal time bias also may have influenced the results because patients undergoing local therapy necessarily survived long enough after recurrence to undergo reassessment, referral, and treatment, whereas those with early progression or deterioration were more likely to remain in the conventional management group.

Across studies, the terms “isolated” and “oligometastatic” recurrence were frequently used to justify local therapy, but definitions and eligibility criteria were heterogeneous and often inadequately reported.^23,49^ Recurrence definitions also varied, with most studies relying on radiologic recurrence, whereas histologic confirmation and biochemical recurrence were inconsistently addressed. Locoregional recurrence was particularly heterogeneous, including the remnant pancreas, pancreatic bed, regional lymph nodes, paraaortic lymph nodes, or perivascular soft-tissue recurrence. Key determinants such as lesion number, disease-free interval, prior systemic therapy exposure, and radiographic response were inconsistently applied. The large number of unreported entries in Table 1 should be interpreted as an important finding rather than merely a limitation of data extraction, underscoring the need for minimum reporting standards to improve reproducibility and strengthen future evidence synthesis.

Several limitations of this study should be acknowledged. Most of the included studies were retrospective and at moderate to serious risk of bias due to confounding and selection bias, particularly within surgical and ablative cohorts. Cohort size-weighted, time-specific survival estimates were provided for context but lacked uncertainty measures and may reflect different contributing studies across time points. For locoregional recurrence, “resection” covered a wide range of procedures, likely reflecting both technical feasibility and tumor biology. For SBRT, the lack of concurrent control groups limited inference regarding comparative effectiveness, and the small number of cohorts precluded robust summaries beyond early time points. A small minority of off-target cases may have been included in some cohorts, precluding uniform classification of recurrence patterns.

Publication bias and small-study effects could not be excluded, and funnel plot assessment was exploratory due to fewer than 10 studies per comparison. In addition, some survival data were obtained by digitizing published Kaplan–Meier curves, which may have introduced minor imprecision. Despite these limitations, this review provides a comprehensive, pattern-based synthesis of post-recurrence outcomes after curative-intent PDAC resection.

In conclusion, this analysis identified site-specific associations between local therapy and longer survival for selected patients with single-site recurrent PDAC. Although current observational evidence does not confirm method-specific efficacy, selected local approaches may benefit patients with biologically limited and technically manageable recurrences. These findings support the need for recurrence pattern-informed prospective comparative studies, including randomized designs where feasible, to define optimal site-tailored strategies. This perspective aligns with ongoing efforts to characterize recurrence patterns and optimize post-recurrence decision-making, including the POCÉMON (Pattern Of reCurrence after pancreatEctoMy and ONcological treatment) studies (NCT07241676), which may help standardize the characterization of recurrence patterns and post-recurrence treatment data, including recurrence timing, disease burden, treatment sequence, and survival time origin for future recurrence pattern-specific comparative studies.

## Supplementary Information

Below is the link to the electronic supplementary material.

Supplementary file 1 (DOCX 25 kb)

Supplementary file 2: Fig. S1. Weighted overall survival after liver recurrence by treatment strategy. **a** Liver resection. **b** Chemotherapy. Three studies contributed to **a** (*n* = 44), and three studies contributed to **b** (*n* = 115). (JPEG 454 kb)

Supplementary file 3: Fig. S2. Weighted survival after lung recurrence by treatment strategy. **a** Post-recurrence survival (PRS) after lung resection. **b** PRS after chemotherapy. **c** Overall survival after lung resection. Contributing studies and total *n* were (**a**) 1 and 2 years: 7 (*n* = 104); 3 years: 6 (*n* = 95), (**b**) 1 year: 3 (*n* = 71); 2 years: 2 (*n* = 63), and (**c**), 4 (*n* = 35). (JPEG 683 kb)

Supplementary file 4: Fig. S3. Weighted survival after locoregional recurrence by treatment strategy. **a** Post-recurrence survival after locoregional resection. **b** Overall survival after stereotactic body radiotherapy (SBRT). **c** Progression-free survival after SBRT. Contributing studies and total *n* were: (**a**) 1 and 2 years: 4 (*n* = 186); 3 years: 3 (*n* = 175), and (**b**, **c**) 3 (*n* = 77). (JPEG 625 kb)

Supplementary file 5: Fig. S4. Risk-of-bias assessment using ROBINS-I. **a** Traffic-light plot of domain-level judgments (D1 confounding, D2 selection, D3 classification of interventions, D4 deviations, D5 missing data, D6 outcome measurement, D7 selective reporting) and overall risk of bias for each study. **b** Summary plot showing proportions at low (*green*), moderate (*yellow*), and serious (*red*) risk by domain and overall. (JPEG 1651 kb)

Supplementary file 6: Fig. S5. Funnel plots for the main comparative analyses. **a** Liver-only recurrence: local therapy versus chemotherapy for overall survival. **b** Lung-only recurrence: resection versus conventional management for post-recurrence survival. **c** Lung-only recurrence: resection versus conventional management for overall survival. **d** Locoregional-only recurrence: resection versus conventional management for post-recurrence survival. All funnel plots were interpreted as exploratory because each comparison included fewer than 10 studies. (JPEG 997 kb)

Supplementary file 7 (DOCX 16 kb)

## Data Availability

All data analyzed in this study were extracted from published articles. The extraction forms and analytic code are available from the corresponding author upon reasonable request.
