# Supplementary material for: Treatment Strategies and Outcomes for Single-Site Recurrent Pancreatic Ductal Adenocarcinoma After Curative-Intent Surgery: Local Therapies are Associated with Longer Survival in Selected Cases: A Systematic Review and Meta-analysis
Source: Ann Surg Oncol. 2026 Jun 16;33(9):8438–47. doi: 10.1245/s10434-026-19939-w (PMC13452759; doi:10.1245/s10434-026-19939-w)
Supplement: Supplementary file 1 [file 10434_2026_19939_MOESM1_ESM.docx]

**Table S1. Characteristics of studies on liver-only recurrence**

| **Author, Year** | **Country** | **Study design** | **Study period** | **Study type** | **Local therapy** | **Comparator** | **No. of patients**  **(liver-only)** |
| --- | --- | --- | --- | --- | --- | --- | --- |
| Schwarz 2020 | International | Retrospective | NR | Comparative | Surgical resection | Chemotherapy | 33 |
| Mitsuka 2020 | Japan | Retrospective | 2005–2015 | Comparative | Surgical resection | Chemotherapy | 17 |
| Shibata 2023 | Japan | Retrospective | 2007–2020 | Comparative | Surgical resection | Chemotherapy | 71 |
| Kim 2025 | Korea | Retrospective | 2006–2018 | Comparative | Surgical resection | Chemotherapy* | 43 |
| Lee 2020 | Korea | Retrospective | 2002–2017 | Comparative | RFA | Chemotherapy | 126 |
| Wang 2023 | China | Retrospective | 2010–2021 | Comparative | RFA | Chemotherapy | 60 |
| NR, not reported; RFA, radiofrequency ablation. | | | | | | | |
| * Outcomes were reported for three groups (surgery, chemotherapy, and best supportive care); for the meta-analysis, only the surgery versus chemotherapy comparison was extracted. | | | | | | | |

**Table S2. Characteristics of studies on lung-only recurrence**

| **Author, Year** | **Country** | **Study design** | **Study period** | **Study type** | **Local therapy** | **Comparator** | **No. of patients**  **(lung-only)** |
| --- | --- | --- | --- | --- | --- | --- | --- |
| Groot 2019 | USA | Retrospective | 2000–2013 | Comparative | Surgical resection | Chemo(radio)therapy | 96 |
| Kurahara 2020 | Japan | Retrospective | 2000–2017 | Comparative | Surgical resection | Chemotherapy | 33 |
| Safi 2021 | Germany | Retrospective | 2007–2016 | Comparative | Surgical resection | Chemotherapy | 17 |
| Mashiko 2021 | Japan | Retrospective | 2006–2018 | Comparative | Surgical resection | Chemotherapy or BSC | 24 |
| Homma 2022 | Japan | Retrospective | 2010–2014 | Comparative | Surgical resection | Chemotherapy or BSC | 117 |
| Stuart 2023 | USA | Retrospective | 2009–2021 | Comparative | Surgical resection | Chemotherapy or RT or BSC | 39 |
| Arnaoutakis 2011 | USA | Retrospective | 2000–2009 | Comparative | Surgical resection | Non-resection | 31 |
| Otsuka 2020 | Japan | Retrospective | 2002–2018 | Comparative | Surgical resection | Non-resection | 48 |
| Shimizu 2020 | Japan | Retrospective | 2008–2016 | Comparative | Surgical resection | BSC | 13 |
| Ilmer 2019 | Germany | Retrospective | 2003–2015 | Descriptive | Surgical resection | NR | 11 |
| BSC, best supportive care; RT, radiotherapy; NR, not reported; USA, United States of America. | | | | | | | |

**Table S3. Characteristics of studies on locoregional-only recurrence**

| **Author, Year** | **Country** | **Study design** | **Study period** | **Study type** | **Local therapy** | **Comparator** | **No. of patients (locoregional-only)** |
| --- | --- | --- | --- | --- | --- | --- | --- |
| Strobel 2013 | Germany | Retrospective | 2001–2009 | Comparative | Surgical resection | Chemo(radio)therapy or BSC | 57 |
| Yamada 2018 | Japan | Retrospective | 2001–2014 | Comparative | Surgical resection | Chemo(radio)therapy or BSC | 114 |
| Kim 2019 | Korea | Retrospective | 2000–2014 | Comparative | Surgical resection | Chemotherapy or BSC | 34 |
| Safi 2021 | Germany | Retrospective | 2007–2016 | Comparative | Surgical resection | Chemotherapy | 28 |
| Comito 2017 | Italy | Retrospective | 2011–2015 | Descriptive | SBRT | NR | 31 |
| Li 2020 | China | Retrospective | 2009–2019 | Descriptive | SBRT | NR | 27 |
| Reddy 2022 | USA | Retrospective | 2012–2018 | Descriptive | SBRT | NR | 19 |
| BSC, best supportive care; SBRT, stereotactic body radiation therapy; NR, not reported; USA, United States. | | | | | | | |
