# Supplementary material for: Treatment Strategies and Outcomes for Single-Site Recurrent Pancreatic Ductal Adenocarcinoma After Curative-Intent Surgery: Local Therapies are Associated with Longer Survival in Selected Cases: A Systematic Review and Meta-analysis
Source: Ann Surg Oncol. 2026 Jun 16;33(9):8438–47. doi: 10.1245/s10434-026-19939-w (PMC13452759; doi:10.1245/s10434-026-19939-w)
Supplement: Supplementary file 2 [file 10434_2026_19939_MOESM7_ESM.docx]

**Supplementary material**

**Search strategy summary (2010–2025)**

| **Database** | **Search Terms / Strategy** | **Results** |
| --- | --- | --- |
| PubMed | ( "pancreatic neoplasms"[MeSH Terms] OR "pancreatic cancer"[Text Word] OR "pancreatic ductal adenocarcinoma"[Text Word] OR "pancreatic adenocarcinoma"[Text Word] OR "pancreatic malignancy"[Text Word] OR PDAC[Text Word] ) AND ( "Neoplasm Recurrence, Local"[MeSH Terms] OR recurrence[Text Word] OR recurrent[Text Word] OR relapsed[Text Word] ) AND ( "Antineoplastic Agents"[MeSH Terms] OR chemotherapy[Text Word] OR systemic therapy[Text Word] OR "Drug Therapy"[MeSH Terms] OR radiotherapy[Text Word] OR "Radiotherapy"[MeSH Terms] OR ablation[Text Word] OR "Ablation Techniques"[MeSH Terms] OR "Surgical Procedures, Operative"[MeSH Terms] OR "Neoplasm, Residual"[MeSH Terms] OR "re-resection"[Text Word] OR "salvage surgery"[Text Word] OR "surgical management"[Text Word] ) AND ( "Treatment Outcome"[MeSH Terms] OR outcome*[Text Word] OR prognosis[Text Word] OR survival[Text Word] ) | 2979 |
| Scopus | ("pancreatic cancer" OR "pancreatic ductal adenocarcinoma" OR PDAC) AND (recurrence OR recurrent OR relapsed) AND (chemotherapy OR radiotherapy OR ablation OR surgery OR resection OR reoperation OR immunotherapy) AND ("treatment outcome" OR prognosis OR survival) | 3389 |
| Cochrane | (pancreatic cancer OR pancreatic ductal adenocarcinoma OR PDAC) AND (recurrence OR recurrent OR relapsed) AND (chemotherapy OR radiotherapy OR ablation OR surgery OR resection OR reoperation OR immunotherapy) AND (survival OR prognosis OR "treatment outcome") | 555 trials /  7 reviews |
